# Metathesis‐Sourced Epoxides in Ring‐Opening Copolymerization: Selective Access to Degradable Polythioesters

**DOI:** 10.1002/marc.70247

**Published:** 2026-02-28

**Authors:** Niracha Tangyen, Bhargav R. Manjunatha, Valerio D'Elia, Alex J. Plajer

**Affiliations:** ^1^ Makromolekulare Chemie Universität Bayreuth Bayreuth Germany; ^2^ Department of Materials Science and Engineering Vidyasirimedhi Institute of Science and Technology, (VISTEC) Payupnai Thailand; ^3^ Department of Biological and Environmental Sciences and Technologies (DiSTeBa) University of Salento, S.P. 6, Lecce-Monteroni Lecce Italy; ^4^ Bayrisches Polymer Institut (BPI) Universität Bayreuth Bayreuth Germany

**Keywords:** metathesis, molybdenum, polythioesters, ring‐opening copolymerization

## Abstract

Ester‐substituted cyclopentene epoxides were synthesized via ring‐closing metathesis and epoxidation, providing selective access to monomers suited for highly controlled ring‐opening copolymerization (ROCOP). The new epoxide enables fully alternating phthalic thioanhydride (PTA)/epoxide ROCOP without scrambling side reactions, yielding well‐defined poly(ester‐alt‐thioester)s with molecular weights up to ∼38 kg/mol after optimization of purity and catalyst preparation. Its performance extends to phthalic anhydride (PA) ROCOP and selective terpolymerizations. Substituent variation confirms the robustness of the cyclopentene scaffold. Comparative degradation studies reveal clear chemical differences between oxygen‐ and sulfur‐containing analogues. These results establish metathesis‐derived cyclopentene epoxides as versatile, accessible monomers for degradable polyesters and polythioesters.

## Graphical abstract

Metathesis‐derived cyclopentene epoxides enable strictly alternating, scrambling‐free ring‐opening copolymerization with phthalic thioanhydride, yielding well‐defined polythioesters with molecular weights up to ∼38 kg/mol. Their performance extends to oxygen‐based anhydrides and selective terpolymerizations. Direct comparisons with all‐oxygen analogues reveal clear, linkage‐dependent degradation behavior, establishing these epoxides as versatile monomers for degradable polymers.

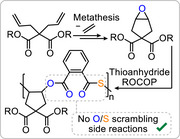

## 1 Introduction

**FIGURE 1:**
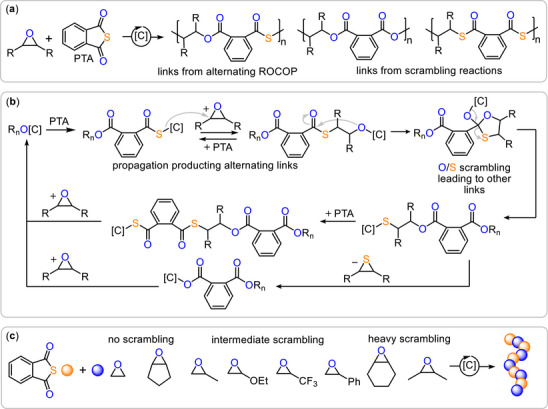
(a) Linkages formed from the ROCOP of PTA with epoxides. (b) Mechanistic showing alternating PTA/epoxide insertion leading to ester‐*alt*‐thioester links and side reactions leading to diester and dithioester links. (c) Previously established selectivity ranking for epoxides in ROCOP with PTA. [C] = Catalyst; R_n_ = polymer chain.

Alternating ring‐opening copolymerization (ROCOP) has become a powerful and versatile method for constructing heteroatom‐containing polymers from combinations of heterocycles and heteroallenes. The most widely explored systems pair oxygen‐based monomers—typically carbon dioxide or cyclic anhydrides and epoxides—to access a broad family of polycarbonates and polyesters with tunable architectures and properties [1, 2, 3]. Among these, phthalic‐anhydride‐derived semi‐aromatic polyesters remain particularly prominent due to their synthetic reliability in the polymerization process [4, 5, 6, 7, 8, 9]. Building on this foundation, ROCOP has more recently been extended into the sulfur domain, enabling access to polythioesters from phthalic thioanhydride (PTA) (see Figure 1a) and to poly(thiocarbonate)s via carbon disulfide (CS_2_) in combination with epoxides [10, 11]. These methodologies unlock the distinct property advantages of sulfur for the field of ROCOP polymers [12, 13, 14, 15, 16, 17, 18, 19, 20, 21, 22, 23, 24, 25, 26, 27, 28, 29, 30].

However, sulfur‐containing ROCOP systems behave fundamentally differently from their all‐oxygen analogues. Instead of clean, perfectly alternating propagation, they frequently suffer from competitive side‐reactions that erode microstructural control [31, 32, 33, 34, 35, 36, 37, 38]. A central issue is oxygen–sulfur scrambling: alkoxide chain ends can undergo backbiting into neighboring polymer links, generating thiolate species that either propagate or undergo further side reactions such as thiirane elimination (see Figure 1b). The latter reaction produces carboxylate chain ends that also propagate. Such deviations from ideal alternation are difficult to suppress and lead to materials with unpredictable composition, inconsistent properties, and limited scalability. Understanding and overcoming these scrambling pathways, therefore, remains a key challenge in the development of well‐defined sulfur‐containing ROCOP materials with predictable properties [39, 40, 41, 42, 43, 44]. In a recent systematic study of PTA/epoxide ROCOP, we identified the structural features required for achieving selective polymerization (see Figure 1) [45]. For example, using epoxides that lead to the formation of primary alkoxide chain‐ends, such as ethylene oxide, led to selective ROCOP as this favours propagation over back‐biting thermodynamically. Furthermore, we found that epoxides capable of forming highly strained transition states during backbiting raise the energetic barrier of scrambling pathways, thereby kinetically favouring clean, alternating propagation. This effect was most pronounced for cyclopentene oxide (CPO), which delivered perfectly alternating poly(ester‐alt‐thioester) under conditions where common epoxides such as propylene oxide or cyclohexene oxide produced scrambled microstructures. However, despite its excellent selectivity, CPO suffered from practical limitations: conversions plateaued below 50%, and achievable molecular weights remained modest (≈10 kg/mol). These shortcomings highlighted the need for improved methodologies that retain the favourable selectivity of the CPO scaffold while enabling higher polymerization performance.

Nevertheless, the mechanistic behaviour of CPO strongly suggested that CPO‐derived epoxides could serve as highly attractive monomers for sulfur‐containing ROCOP. Unlike monosubstituted epoxides—where numerous variants are readily available—structurally diverse CPO derivatives are much less accessible. Motivated by this synthetic bottleneck, we sought a synthetic route to functionalised cyclopentene frameworks. We identified alkene metathesis of allyl‐functionalized malonates as an efficient strategy to access substituted cyclopentenes, which can then be cleanly epoxidized. This provides a straightforward and flexible entry point to ester‐substituted CPO derivatives, offering handles for further structural variation and ultimately motivating the studies described herein.

## 2 Results and Discussion

**FIGURE 2:**
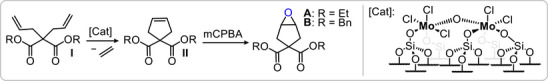
Synthetic scheme yielding cyclopentene oxides A and B.

As shown in Figure 2, accessing cyclopentenes II from I via ring‐closing metathesis (RCM) represents the first key step toward synthesizing cyclopentene oxides (see Section S2). The RCM step toward II is most typically carried out using ruthenium‐based Grubbs‐type olefin metathesis catalysts [46]. In the search for more sustainable alternatives to the use of precious ruthenium, alkylidene complexes of more abundant metals (Mo, V) have also been reported [47, 48]. However, such complexes are not commercially available and would require multistep synthesis under air‐sensitive conditions, thus limiting their suitability for the multigram synthesis of monomers as required for the polymerization in our study. Through a surface‐organometallic chemistry approach [49, 50, 51], we recently evaluated simple silica‐supported molybdenum oxychlorides, in the presence of SnMe_4_ as the activator, as a readily available option for the ethenolysis of fatty acids [52]. These materials avoid the use of precious ruthenium or the multistep synthesis of metal alkylidene complexes. Using this catalyst system, we evaluated a range of conditions to optimize the RCM step for the ethyl‐version of I. At 80 °C and 2.5 mol% catalyst loading (Table 1 run 1), the reaction reached just over 50% conversion after 21 h. Increasing the catalyst loading (runs 2 and 3) or the amount of activator (run 4) significantly improved conversion, and extending the reaction time (runs 5–7) ultimately delivered quantitative turnover. Under these optimized conditions, even reduced temperatures (runs 8 and 9) maintained high activity, achieving >90% conversion after 21 h. Importantly, the methodology proved scalable to the multi‐gram quantities required for subsequent polymerization studies. Epoxidation of the metathesis product then cleanly afforded epoxide A.

**TABLE 1:** Screening the ring‐closing metathesis conditions employing 0.5mL of I.

Run	R	Cat. [mol%, mg]	SnMe_4_ [µL]	*T* [°C]	*t* [h]	Conv. [%]
1	Et	2.5, 75	100	80	21	52
2	Et	3.3, 100	100	80	24	60
3	Et	5.0, 150	100	80	24	96
4	Et	5.0, 150	200	80	10	95
5	Et	5.0, 150	200	80	15	98
6	Et	5.0, 150	200	80	18	98
7	Et	5.0, 150	200	80	21	>99
8	Et	5.0, 150	200	70	21	95
9	Et	5.0, 150	200	60	21	92
10	Bn	10, 200	200	100	24	17

With epoxide A in hand, we next examined its ROCOP reactivity with PTA (see Sections S3 and S4), motivated by the promising behavior previously observed for cyclopentene oxide scaffolds. We initially screened several catalysts, including bimetallic systems (Table 2 run 1), which are an emerging class of high‐performing catalysts for ring‐opening (co)polymerization [53, 54, 55, 56, 57, 58, 59, 60]. Particular attention was given to the Aluminium(III)–Rubidium(I) complex LAlRb(OAc)_2_, which has demonstrated high activity in PTA/epoxide ROCOP. Under standard conditions (1:100:100 catalyst:PTA:A at 80 °C), ROCOP proceeded rapidly, reaching 82% PTA conversion within 0.5 h and producing a viscous reaction mixture. Precipitation from methanol cleanly isolated the polymer.

**TABLE 2:** ROCOPs investigated in this study.

	Catalyst	Monomer	Ratio	*T* [°C]	*t* [h]	Conv. [%]^a^	*M*_n_ [kg/mol] (*Đ*)^b^
1	LAlRb(OAc)_2_ ^d^	PTA/A^c^	1:100:100	80	0.5	82	8.3 (1.2)
2	BEt_3_:PPNCl	PTA/A^c^	1:100:100	80	1.5	80	10.8 (1.4)
3	LAlRb(OAc)_2_ ^d^	PTA/A^c^	1:500:500	80	2.0	68	13.6 (1.3)
4	LAlRb(OAc)_2_ ^d^	PTA/A	1:100:100	80	0.5	88	15.1 (1.2)
*5*	LAlRb(OAc)_2_ ^d^	PTA*/A	1:100:100	80	0.4	92	16.2 (1.2)
6	LAlRb(OAc)_2_ ^e^	PTA*/A	1:100:100	80	0.4	86	17.9 (1.3)
7	LAlRb(OAc)_2_ ^e^	PTA*/A	1:500:1000	100	0.6	88	37.8 (1.3)
8	LAlRb(OAc)_2_ ^e^	PTA*/A	1:1000:2000	100	2.0	84	37.5 (1.2)
9	LAlRb(OAc)_2_ ^e^	PTA*/CPO	1:500:1000	100	3.0	74	20.8 (1.4)
10	LAlRb(OAc)_2_ ^e^	PA/A	1:500:1000	100	3.0	89	30.4 (1.3)
11	LAlRb(OAc)_2_ ^e^	CS_2_/A	1:1000:500	100	0.5	54^f^	12.6 (1.3)
12	LAlRb(OAc)_2_ ^e^	PTA/B^g^	1:500:500	100	0.5	90	12.0 (1.3)
13	LAlRb(OAc)_2_ ^e^	PA/B^g^	1:500:500	100	7.0	62	9.1 (1.3)

Polymerizations were conducted with epoxide, which was distilled two times with CaH_2_ and NaH. PTA was recrystallized three times from hexane before sublimation. PTA* was recrystallized four times from hexane before sublimation.

^a^ Relative integral of aromatic resonances corresponding to the polymer in the normalized ^1^H NMR spectrum of the crude reaction mixture.

^b^ Determined by GPC (gel permeation chromatography) measurements conducted in THF, using narrow polystyrene standards to calibrate the instrument.

^c^ Epoxide was distilled only once with CaH_2_.

^d^ Catalyst was synthesized using methanol as a solvent.

^e^ Catalyst was synthesized using acetonitrile as a solvent.

^f^ Conversion of epoxide as determined by ^1^H NMR spectrum of crude reaction mixture.

^g^ Epoxide was distilled twice over CaH^2^.

**FIGURE 3:**
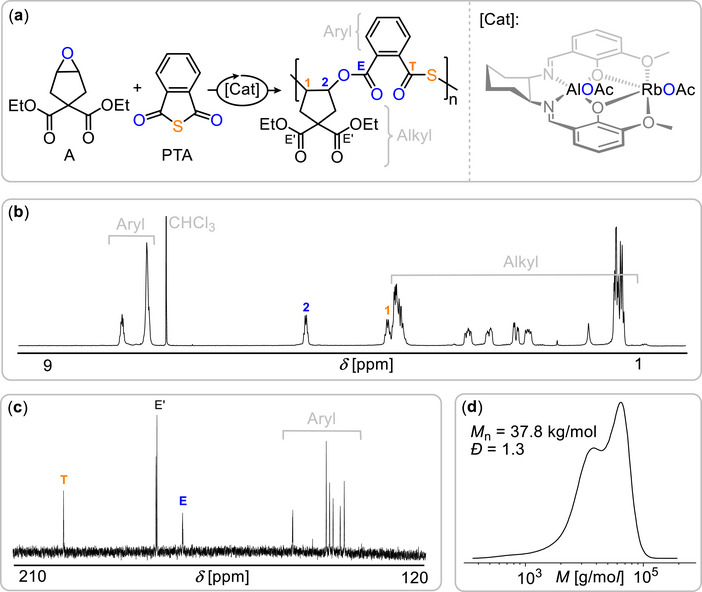
(a) Synthetic scheme for PTA/A ROCOP. (b) ^1^H NMR (CDCl_3_, 300 MHz), (c) ^13^C NMR (CDCl_3_, 126MHz) spectrum and (d) GPC curve corresponding to the polymer of Table 1 run 7.

^1^H and ^13^C NMR spectroscopy confirmed the formation of a strictly alternating poly(ester‐*alt*‐thioester) microstructure (see Figure 3). The characteristic quaternary carbonyl resonances for the ester and thioester units appeared cleanly (192 and 165 ppm, respectively), and their near‐identical relaxation times, as established in our prior contribution [45], enabled direct quantification by integration. A 1:1 integrative ratio of ester to thioester signals was observed, with no minor carbonyl resonances attributable to scrambled linkages. In addition, the tertiary CH signals of the ring‐opened cyclopentyl units adjacent to each carbonyl environment also appeared in a 1:1 integrative ratio. Together, these features unequivocally demonstrate perfectly alternating propagation and the complete suppression of O/S scrambling, consistent with previous observations for cyclopentene‐derived epoxides.

Gel permeation chromatography (THF, polystyrene calibration) revealed an apparent number‐average molecular weight of 8.3 kg/mol with a narrow dispersity (*Đ* = 1.2).

The obtained molecular weight is considerably lower than the theoretical value of ∼30 kg/mol, which we attribute to chain‐transfer processes initiated by protic impurities in the epoxide. Such impurities are well‐known to limit molecular weights in sulfur‐containing ROCOP systems [61, 62].

Accordingly, matrix‐assisted laser desorption/ionization time‐of‐flight (MALDI‐TOF; see Section S6) mass spectrometry of a low‐molecular‐weight polymer allowed us to identify distributions corresponding to polymers formed via chain transfer with partially hydrolyzed epoxides bearing one CO_2_Et and one CO_2_H group. After ring opening, these species can initiate the formation of two polymer strands, effectively acting as bifunctional initiators. Consistent with this interpretation, the GPC traces of the polymer samples presented in this study are bimodal, corresponding to two overlapping distributions of chains formed either via initiation with monofunctional and bifunctional initiators or via chain transfer with monofunctional and bifunctional chain‐transfer agents. To confirm this hypothesis, we performed comparative runs in which an excess (20 equiv; see Section S7) of the bifunctional chain‐transfer agent 1,4‐benzenedimethanol was added, and indeed observed the formation of an almost monomodal distribution, as predominantly bifunctional chains were formed. However, it cannot be excluded that transesterification processes also contribute to the multimodality of the distributions. Furthermore, batch‐to‐batch variability in the level of impurities leading to chain transfer may affect the ratio of the overlapping distributions, making it difficult to control.

Consistent with this interpretation, switching to another catalyst system, BEt_3_/PPNCl (run 2) popular in ROCOP, produced polymers with similarly reduced molecular weights, albeit at a much slower polymerization rate [63]. Furthermore, this catalyst produces a scrambled, oxygen‐enriched polymer as evident in the ^1^H NMR spectrum that features additional aromatic and tertiary CH signals compared to the selective copolymer produced by LAlRb(OAc)_2_.

Attempts to increase molecular weight by simply reducing the catalyst loading employing LAlRb(OAc)_2_ (run 3) led to only modest improvements. We therefore turned to a more systematic evaluation of monomer purity, which—while generally recognized as critical in ROCOP—has been less thoroughly explored in sulfur‐containing systems due to the interference of scrambling side‐reactions. The PTA/A system, where scrambling is completely suppressed by monomer design, provides a unique platform to isolate and study these effects. In the initial experiments, epoxide A had been purified only once by distillation over CaH_2_. Subjecting A to an additional purification step via distillation over NaH (run 4) nearly doubled the molecular weight under otherwise identical conditions. Further purifying PTA by an additional recrystallization (run 5) led to only incremental improvements, indicating that epoxide purity is the dominant factor in determining molecular weight in this system.

The previously reported synthesis of LAlRb(OAc)_2_ employed methanol as the reaction solvent for preparation [53]. Despite careful drying, residual MeOH could remain coordinated to the metal centers or hydrogen‐bonded to the co‐ligands. Such bound alcohols, much like protic impurities in the monomers, are potential chain‐transfer agents and can therefore limit achievable molecular weights. To eliminate this source of contamination, we modified the catalyst preparation by replacing MeOH with the non‐protic solvent acetonitrile. Using this refined catalyst (run 6) led to a subtle increase in molecular weight compared to the original procedure, confirming the sensitivity of the system to adventitious alcohols.

When combined with the improved epoxide and anhydride purification described above, these adjustments collectively more than doubled the *M*_n_ relative to run 1 at comparable conversion. Further optimization by decreasing the catalyst loading (runs 7 and 8) and employing an excess of epoxide to reduce viscosity and improve turnover enabled molecular weights up to 37.8 kg/mol (*Đ* = 1.2) [45]. This represents a more than threefold improvement over our previous results on cyclopentene oxide. Reducing the catalyst loading beyond this point offered no additional benefit. Overall, these results demonstrate that the newly developed epoxide A is a robust monomer for PTA ROCOP and is capable of delivering well‐defined polymers with high molecular weights when monomer and catalyst purity are properly controlled.

Naturally, the question arises of how the ROCOP of A compares to that of cyclopentene oxide (CPO) with the same catalyst, as previous studies employed the combination of Cr(III) with K(I) within a similar yet distinct ligand framework. Employing LAlRb(OAc)_2_, PTA/CPO ROCOP proceeded at a rate similar to that of PTA/A polymerization (run 9). NMR analysis again confirmed clean formation of a poly(ester‐*alt*‐thioester) microstructure without any detectable scrambling, reinforcing the suitability of the CPO scaffold for highly selective PTA ROCOP. Interestingly, compared to A, PTA/CPO ROCOP was at least 4 times slower, while furthermore only yielding a polymer with roughly half the molecular weight, revealing an overall superior performance of our new metathesis‐sourced epoxide.

**FIGURE 4:**
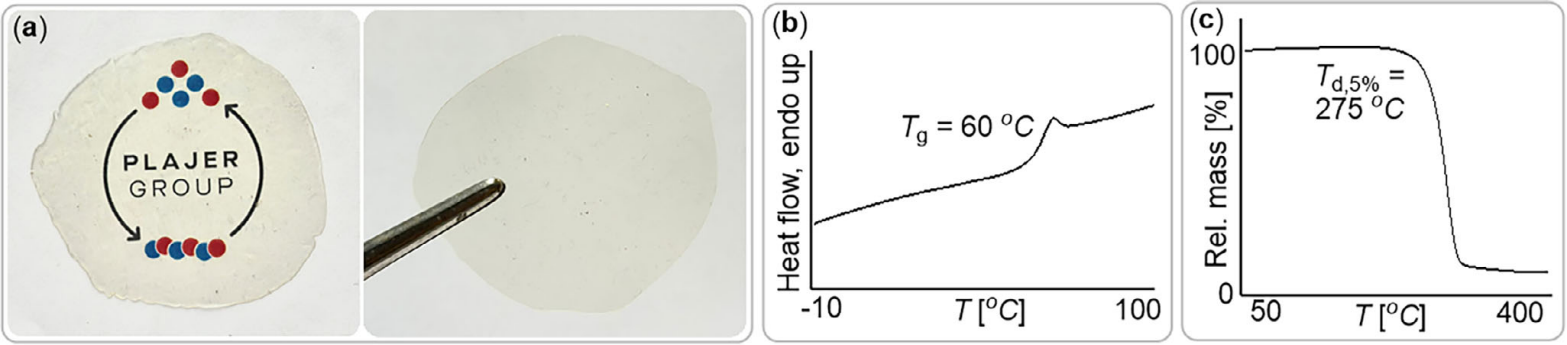
(a) Compression molded polymer film, (b) DSC data (second heating) and (c) TGA data of copolymer corresponding to Table 1 run 7.

Furthermore, the resulting materials showed distinct thermal behavior: while the PTA/A copolymer displayed a glass transition temperature of *T*_g_ = 60°C (see Figure 4), the PTA/CPO material exhibited a substantially higher *T*_g_ of 87°C. This difference highlights that introducing flexible alkyl ester substituents into the polymer backbone—via monomer A—offers an effective strategy for tuning the thermal properties of ROCOP‐derived thioesters.

We next evaluated the reactivity of A with other anhydrides. Substituting PTA with phthalic anhydride (PA) also resulted in efficient ROCOP (run 10), though at a noticeably reduced rate: under conditions identical to run 7, the PTA system reached comparable conversions in less than one‐third of the time. The PA/A polymer was isolated as a clean polyester with a number‐average molecular weight of *M*_n_ = 30.4 kg/mol (*Đ* = 1.3). In terms of thermal properties, the PTA/A and PA/A materials showed broadly similar behavior, with the PTA/A copolymer exhibiting a decomposition onset of *T*_d,5%_ = 275 °C by TGA (see Figure 3D). For comparison, the all‐oxygen PA/A variant at comparable molecular weight displayed a *T*_g_ = 62°C, which is very similar to the PTA variant and a *T*_d,5%_ = 314°C, indicating a modest enhancement in thermal stability relative to the corresponding thioester analogue.

As expected, O/S scrambling is not a concern in the PA/A system, forming a clean polyester with a quaternary carbonyl resonance at 166 ppm from the polymer backbone, since neither monomer contains sulfur. The situation is markedly different for CS_2_/epoxide ROCOP, where O/S exchange is among the most pronounced of any ROCOP system reported to date. CS_2_/A ROCOP (run 11) did proceed efficiently, reaching 54% epoxide conversion after 0.5 h and yielding a polymer with an apparent *M*_n_ = 12.6 kg/mol (*Đ* = 1.3). However, the crude NMR spectrum showed formation of small‐molecule cyclic dithiocarbonate byproducts. Unlike the PTA system, substantial scrambling was also observed. Although the alternating dithiocarbonate linkage remained the dominant repeat unit, the ^1^H NMR spectrum showed multiple additional resonances corresponding to the formation of mixed (thio)carbonate linkages. ^13^C NMR analysis further confirmed that monothiocarbonate links, corresponding to a carbonyl resonance at 168 ppm, were the most prevalent scrambled species alongside the unscrambled dithiocarbonate links, corresponding to a carbonyl resonance at 212 ppm.

The CS_2_/A copolymer exhibited a significantly lower *T*_g_ = 37°C and a reduced thermal stability (*T*_d,5%_ = 165°C) compared to the PA/A and PTA/A materials. The reduced glass transition temperature is consistent with the increased flexibility of heterocarbonate linkages relative to the semiaromatic (thio)ester backbones. The diminished thermal stability likely reflects the ready access to depolymerization pathways leading to cyclic carbonates, which lowers the overall activation barrier for thermal degradation.

Beyond variation of the ROCOP comonomer, the alkyl ester substituents on the epoxide itself offer an additional opportunity to tune polymer structure and properties. To demonstrate this, we synthesized the dibenzyl‐ester–substituted analogue of II using the metathesis strategy established in Table 1 for later epoxidation. Although RCM of the dibenzyl precursor did occur (Table 1 run 10), even elevated temperatures afforded only modest conversion (<20%). For preparative purposes, we therefore turned to a Grubbs catalyst, which cleanly and quantitatively delivered the desired alkene. Subsequent epoxidation furnished the dibenzyl‐substituted cyclopentene oxide B. In contrast to epoxide A, compound B could not be purified via NaH, likely due to the lability of the benzyl groups; thus, material purified only by CaH_2_ distillation was used for ROCOP. Despite this limitation, PTA/B ROCOP proceeded smoothly (Table 2 run 12), reaching 90% conversion at 100 °C under 500:500 monomer loading with respect to LAlRb(OAc)_2_. The resulting polymer had *M_n_* = 12.0 kg/mol (*Đ* = 1.3). As with epoxide A, NMR spectroscopy confirmed a clean poly(ester‐*alt*‐thioester) microstructure with no evidence of scrambling, underscoring the robustness of the cyclopentene oxide scaffold toward suppressing side‐reactions. The PTA/B copolymer exhibited a glass transition temperature of 43°C (DSC) and a thermal decomposition onset of 268°C (TGA), demonstrating that modification of the ester substituent provides a viable handle for tuning material properties while maintaining excellent sequence fidelity.

Having established A as a versatile comonomer for various ROCOP systems, we next explored its behavior in terpolymerizations. A three‐component terpolymerization of PTA, PA, and A (250:250:1000 relative to catalyst) was conducted, and aliquots were taken periodically to follow monomer consumption. ^1^H NMR analysis revealed the formation of a gradient‐type structure, with PA being consumed preferentially before PTA. Also in this case, PTA/A ROCOP occurred without any scrambling. After near‐complete consumption of both anhydrides, the resulting polymer displayed *M*_n_ = 30.5 kg/mol (*Đ* = 1.2), a *T*_g_ of 62°C, and a *T*_d,5%_ of 292 °C, closely resembling the values of the corresponding binary copolymers.

We then attempted a terpolymerization involving CS_2_, PTA, and A (500:250:500 relative to catalyst) at 80 °C. As in the anhydride system, PTA was consumed first, but only limited incorporation of thiocarbonate repeat units from CS_2_ ROCOP was observed before polymerization ceased. The final material consisted of approximately 80% PTA/A and 20% CS_2_/A repeat units, as determined by integration of the ^1^H NMR spectrum of the isolated polymer. This block‐like polymer exhibited *M*_n_ = 35.8 kg/mol (*Đ* = 1.3), *T*_g_ = 58°C, and *T*_d,5%_ = 228°C. The reduced thermal stability relative to the standalone PTA/A copolymer is consistent with the presence of thermally labile, thiocarbonate linkages.

Importantly, this stepwise polymerization—PTA incorporation followed by limited CS_2_ incorporation—stands in stark contrast to our previously reported PTA/CS_2_/epoxide ring‐opening depolymerization (ROTERP), where all three monomers combine in a sequence‐selective manner to form a poly(ester‐*alt‐* ester‐*alt*‐trithiocarbonate) repeat unit [64, 65, 66]. That mechanism relies on O/S scrambling, and its suppression by the cyclopentene oxide scaffold in *A* directs the system away from ROTERP‐type behavior and instead toward block formation. Similar behavior was observed when PA was used in place of PTA: PA polymerized first, and only negligible CS_2_ incorporation occurred afterward.

**FIGURE 5:**
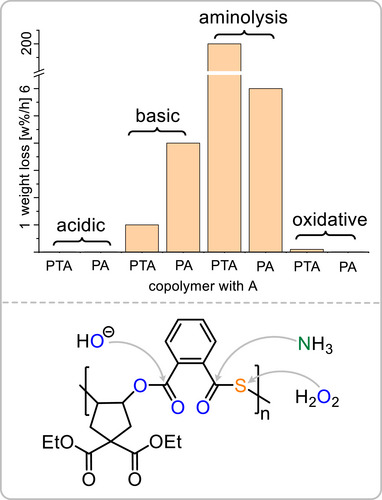
Degradation rate of a copolymer of A with PTA and PA under different conditions and derived preferential reactivities.

The highly controlled copolymerization of PTA with A provides a rare opportunity to directly assess how a single sulfur atom in the repeat unit influences the degradability of the resulting material (see Section S5). Although we previously showed that sulfur‐containing polymers can undergo degradation under a range of conditions [67], a direct comparison with the all‐oxygen analogue was limited. Conveniently, the PTA/A and PA/A copolymers prepared in this study possess similar molecular weights and, owing to the selective ROCOP enabled by the cyclopentene oxide scaffold, differ only by the presence or absence of the thioester linkage. This structural simplicity allowed us to systematically compare their degradation behavior (see Figure 5).

Under acidic conditions (aqueous HCl, 40°C, 7 days), both materials were essentially stable, showing negligible mass loss. In contrast, both polymers were susceptible to basic hydrolysis in aqueous NaOH. The all‐oxygen polyester degraded completely (>99 wt% loss) within 24 h, whereas the sulfur‐containing analogue required approximately four times longer to reach full degradation.

A strikingly different trend emerged when ammonia was used as a base instead of NaOH. Aminolysis proceeded much more rapidly for the sulfur‐containing polymer, which fully degraded within 30 minutes, whereas the all‐oxygen analogue required 17 hours under identical conditions. This behavior indicates a higher susceptibility of thioester to nucleophilic attack by amines compared to esters.

Finally, oxidative degradation was investigated using aqueous hydrogen peroxide. The polyester exhibited no appreciable degradation, while the thioester‐containing material showed 22 wt% mass loss after seven days. This is consistent with oxidation of the sulfur center to sulfoxide or sulfone species, which increases hydrophilicity and thereby facilitates subsequent hydrolytic cleavage.

As shown in Figure 5, it emerges that thioester bonds are more susceptible to degradation via aminolysis and oxidation, while ester bonds are more prone to attack by hydroxide nucleophiles.

## 3 Conclusions

In conclusion, metathesis‐derived cyclopentene oxides enable highly controlled ROCOP, providing strictly alternating poly(ester‐*alt‐*thioester) with no O/S scrambling. Epoxide A is readily accessed via selective RCM/epoxidation and delivers molecular weights up to ∼38 kg/mol when monomer and catalyst purity are optimized. It also performs reliably in PA ROCOP and terpolymerizations, whereas CS_2_ systems continue to scramble. Direct comparison of sulfur‐ and oxygen‐containing analogues reveals clear differences in degradation behaviour, underscoring the distinct chemical reactivity imparted by thioester units. Overall, cyclopentene epoxides provide an accessible platform for designing degradable polyesters and polythioesters with well‐defined microstructures.

## Conflicts of Interest

The authors declare no conflict of interest.

## Supporting information




**Supporting File**: marc70247‐sup‐0001‐SuppMat.docx.

## Data Availability

The data that support the findings of this study are available in the supplementary material of this article.
