# Supplementary material for: Metathesis‐Sourced Epoxides in Ring‐Opening Copolymerization: Selective Access to Degradable Polythioesters
Source: Macromol Rapid Commun. 2026 Feb 28;47(11):e70247. doi: 10.1002/marc.70247 (PMC13238326; doi:10.1002/marc.70247)
Supplement: Supplementary file 1 — Supporting File: marc70247‐sup‐0001‐SuppMat.docx. [file MARC-47-e70247-s001.docx]

**Supporting information**

Metathesis-sourced Epoxides in Ring-Opening Copolymerization:

Selective Access to Degradable Polythioesters

**Table of Contents**

**Section S1:** General methods 3

General synthesis of PTA 3

General synthesis of LAlRb(OAc)_2_ 4

**Section S2:** Ring-closing metathesis and Epoxidation

General synthesis ring-closing metathesis (RCM) compounds 6

General synthesis **A** and **B** 7

^1^H NMR, ^13^C NMR and conversion calculation 8

**Section S3**: Polymerization

General polymerization 20

ROCOP and ROTERP 20

^1^H NMR, ^13^C NMR, ^1^H-^13^C HSQC and ^1^H-^13^C HMBC 21

**Section S4:** GPC, DSC and TGA characterization 64

**Section S5:** Degradation studies 70

**Section S6:** MALDI-TOF 71

**Section S7:** Copolymer with chain transfer agent (CTA) 72

**References** 73

**Section S1: General methods**

Solvents and reagents were obtained from commercial sources and used as received unless stated otherwise. If “dried solvents” were used these were obtained by different procedures. Epoxide compounds were dried with different procedures found in **section** **S3.1**. Phthalic thioanhydride (PTA) and Phthalic anhydride (PA) were described as a protocol in **section** **S1.1**. Carbon disulfide (CS_2_) was dried with calcium hydride before distillation, then degassed and stored in a glove box filled with argon.

Nuclear Magnetic Resonance (NMR) spectra were recorded by using Bruker Advance 300, 500 and 600 MHz spectrometers. 1H and 13C{1H} chemical shifts are referenced to the residual proton resonance of the deuterated solvents. Thermogravimetric Analysis (TGA) data was measured using a Mettler Toledo STARe System ‘’TGA/DSC 3+’’. Differential scanning calorimetry (DSC) was measured on a Mettler Toledo ‘’STARe System DSC 3+’’ at a heating rate of 10.0 K/min. The molecular weight and polydispersity of the polymers were determined by a Waters 515 Gel permeation chromatography (GPC) instrument equipped with two linear PLgel columns (Mixed-C) following guard column and a differential refractive index detector using tetrahydrofuran as the eluent at a flow rate of 1.0 mL/min at 30 °C and a series of narrow polystyrene standards for the calibration of the columns. Each polymer sample was dissolved in HPLC-grade THF (2 mg/mL) and filtered through a 0.20 μm porous filter frit prior to analysis. MALDI-TOF spectra was recorded with a MALDI-TOF Spectrometer Bruker Autoflex Max by using 2,5-dihydroxybenzoic acid (DHB) as matrix and sodium trifluoroacetate (NaTFA) as salt.

**S1.1 General synthesis of PTA protocol:**

PTA was adapted according to the literature.^2^ PA (0.3 mol, 1.0 equiv) was dissolved in 600 mL THF and an aqueous Na_2_S∙9H_2_O (0.2 mol, 0.7 equiv) solution was added slowly. The resulting mixture was stirred at room temperature, and the reaction progress was monitored by NMR analysis. After all PA was consumed, THF was removed in vacuum, and the aqueous phase was extracted three times with DCM and dried over MgSO_4_. The crude thioanhydride was recrystallized three times from hexane and further purified by sublimation and stored inside a glovebox.

**S1.2 General synthesis of LAlRb(OAc)_2_** **protocol:**

**LAlRb(OAc)_2_ complex** was synthesized according to an adapted literature procedure.^1^

Under an argon atmosphere, **L^cy^AlOAc** (300 mg, 1 equivalent) was added to 10 mL of tetrahydrofuran (THF). Subsequently, trimethylaluminum (783 $\mu$L, 1 equivalent) was slowly added to the solution. The reaction mixture was then removed from the glovebox to stir. After 2 h, a precipitate formed. Acetic acid (90 $\mu$L acetic acid, 2 equivalents) was then added to the solution, and it was stirred overnight. The solid product was collected by centrifugation, washed twice with toluene, and then with pentane.

**L^Cy^AlOAc** (200 mg, 1 equivalent) and **RbOAc** (62 mg, 1 equivalent) were dissolved in 250 mL of dried acetonitrile in a flask equipped with a magnetic stirrer bar. The mixture was heated to 85 °C and briefly heated with a heat gun to facilitate dissolution, then stirred overnight. Acetonitrile was removed by evaporation under vacuum. The resulting catalyst compound was washed three times with diethyl ether and then dried in a vacuum oven at 50 °C for 3 days.

1. ^1^H NMR spectrum (300 MHz, Methanol-d_4_) of LAlRb(OAc)_2_

**Section S2: Ring-closing metathesis and Epoxidation**

**S2.1 General synthesis ring-closing metathesis (RCM) compound protocol:**

**activation of BM-20%w/w MoOCl_4_@SiO_2-250_ catalyst**

Under inert atmosphere, the precatalytic **BM-20%w/wMoOCl_4_@SiO_2-250_** was synthesized following the literature.^3^ Before use, precatalyst will be activated by dissolving it in dichloromethane carried out in 150 mL pressure vessel equip with magnetic stir. Tetramethyl tin (SnMe_4_) activator was then added, and the mixture was stirred at 530 rpm for 1 h at room temperature.

**diethyl cyclopent-3-ene-1,1-dicarboxylate (II,Et)**

After the catalytic activation process, diethyl allymalonate was added to the solution. The reaction was removed from the glovebox and carried out under an air atmosphere. The mixture was stirred at 530 rpm at the desired temperature for 24h. The reaction mixture was then passed through a vacuum filtration system. The metathesis crude solution was extracted sequentially with 1 M potassium fluoride (KF) solution (1 time) and then with saturated sodium chloride (NaCl) solution (1 time) to remove tin impurities. The purified metathesis solution was then evaporated under vacuum to collect the ring-closing metathesis product. **II,Et** isolated yield = 90%

^1^H NMR (600 MHz, Chloroform-*d*) δ 5.60 (s, 2H), 4.19 (q, *J* = 7.1 Hz, 4H), 3.01 (s, 4H), 1.25 (t, *J* = 7.1 Hz, 6H).

^13^C NMR (151 MHz, Chloroform-*d*) δ 172.2, 127.8, 61.5, 58.8, 40.9, 14.0.

**dibenzyl cyclopent-3-ene-1,1-dicarboxylate (II,Bn)**

The starting material for the ring-closing metathesis, dibenzyl 2,2-diallylmalonate, was synthesized according to an adapted literature procedure.^4^ Dibenzyl malonate (5 mL, 20 mmol) was added to a mixture of potassium carbonate (11.6 g, 84 mmol) and tetrabutylammonium hydrogen sulfate (1.1 g, 3.2 mmol) dissolved in acetonitrile. Ally bromide (3.5 ml, 41 mmol) was then added, and the mixture was refluxed for 5 days. After reaction was finished, distilled water was added to crude reaction, which was then extracted three times with diethyl ether. The organic phase was dried with magnesium sulfate and evaporated under vacuum.

Under an inert atmosphere, the dibenzyl 2,2-diallylmalonate was dissolved in dichloromethane in a pressure vessel equipped with a magnetic stir bar. Grubbs catalyst, 1^st^ generation, was then added. The reaction was removed from the glovebox and carried out under an air atmosphere, stirring at 530 rpm at 80 °C for 24 h.

After the reaction was finished, the crude product was evaporated under vacuum. DMSO was then added to deactivate the Grubbs catalyst by stirring overnight. The crude reaction mixture and catalyst were then separated by flash column chromatography using a hexane:ethyl acetate (10:1) solvent system. **II,Bn** isolated yield = 80%

^1^H NMR (600 MHz, Methanol-*d*_4_) δ 7.37 – 7.28 (m, 10H), 5.64 (ddt, *J* = 18.6, 8.9, 7.4 Hz, 2H), 5.12 (s, 4H), 5.08 – 5.04 (m, 4H), 2.66 (d, *J* = 7.4 Hz, 4H).

^13^C NMR (151 MHz, Methanol-*d*_4_) δ 171.71, 135.70, 128.16, 127.92, 127.77, 127.34, 66.92, 58.89, 40.42.

**S2.2 General synthesis diethyl 6-oxabicyclo [3.1.0]hexane-3,3-dicarboxylate (A) and** **dibenzyl 6-oxabicyclo[3.1.0]hexane-3,3-dicarboxylate (B)**

The ring-closing metathesis product (1 equiv) was dissolved in 250 mL of dichloromethane and stirred at 530 rpm in an ice bath. Meta-chloroperoxybenzoic acid (m-CPBA) (1.2 equiv) was then added to the solution, and stirring continued for 1.5 h. After 1.5 h, the ice bath was removed, and stirring continued for a further 24 h at 530 rpm at room temperature. Both ring-closing metathesis compounds provide more than 99% conversion.

The crude reaction mixture was sequentially extracted three times with 1 M sodium sulfite solution, three times with saturated sodium bicarbonate solution, and finally once with saturated sodium chloride solution. The combined extracts were dried with magnesium sulfate. The filtrate was collected by filtration, and the solvent was then removed in vacuo to afford the final product. **A** isolated yield = 88% and **B** isolated yield = 88%.

**diethyl 6-oxabicyclo [3.1.0]hexane-3,3-dicarboxylate (A)**

^1^H NMR (600 MHz, Chloroform-*d*) δ 4.23 – 4.12 (m, 4H), 3.51 (s, 2H), 3.02 (d, *J* = 14.4 Hz, 2H), 2.19 (d, *J* = 14.5 Hz, 2H), 1.24 (dt, *J* = 21.8, 7.1 Hz, 6H).

^13^C NMR (151 MHz, CDCl_3_) δ 171.2, 62.0, 55.8, 55.5, 35.89, 14.0.

**dibenzyl 6-oxabicyclo[3.1.0]hexane-3,3-dicarboxylate (B)**

^1^H NMR (300 MHz, Chloroform-*d*) δ 7.40 – 7.12 (m, 10H), 5.11 (d, *J* = 14.0 Hz, 4H), 3.53 (s, 2H), 3.08 (d, *J* = 14.5 Hz, 2H), 2.22 (d, *J* = 14.5 Hz, 2H).

^13^C NMR (151 MHz, CDCl_3_) δ 170.8, 135.6, 128.6, 128.4, 128.1, 127.9, 67.5, 55.9, 55.5, 36.0.

**S2.3 ^1^H and ^13^C NMR and Conversion calculation**

**Diethyl cyclopent-3-ene-1,1-dicarboxylate (II,Et)**

1. ^1^H NMR spectrum (600 MHz, Chloroform-d) of **I,Et** ring-closing metathesis crude reaction catalyzed by BM-20%-MoOCl_4_@SiO_2-250_ (table 1, run 1)

conversion = $\frac{\boldsymbol{(}\boldsymbol{H}_{\boldsymbol{c}}\boldsymbol{+}\boldsymbol{H}_{\boldsymbol{c'}}\boldsymbol{+}\boldsymbol{H}_{\boldsymbol{d}}\boldsymbol{+}\boldsymbol{H}_{\boldsymbol{d'}}\boldsymbol{)}}{\left( \boldsymbol{H}_{\boldsymbol{c}}\boldsymbol{+}\boldsymbol{H}_{\boldsymbol{c'}}\boldsymbol{+}\boldsymbol{H}_{\boldsymbol{d}}\boldsymbol{+}\boldsymbol{H}_{\boldsymbol{d'}} \right)\boldsymbol{+(}\boldsymbol{H}_{\boldsymbol{c}}\boldsymbol{+}\boldsymbol{H}_{\boldsymbol{c'}}\boldsymbol{+}\boldsymbol{H}_{\boldsymbol{d}}\boldsymbol{+}\boldsymbol{H}_{\boldsymbol{d'}}\boldsymbol{)}}$x100

conversion = $\frac{\boldsymbol{(}\boldsymbol{1}\boldsymbol{.}\boldsymbol{00}\boldsymbol{)}}{\left( \boldsymbol{1}\boldsymbol{.}\boldsymbol{00} \right)\boldsymbol{+(}\boldsymbol{0}\boldsymbol{.}\boldsymbol{93}\boldsymbol{)}}$x100

conversion = 52 %

1. ^1^H NMR spectrum (600 MHz, Chloroform-*d*) of isolated **I,Et** before treated with KF

1. ^1^H NMR spectrum (600 MHz, Chloroform-*d*) of isolated **II,Et** after KF extraction

1. ^13^C NMR (151 MHz, Chloroform-*d*) of isolated **II,Et** after KF extraction

**Dibenzyl cyclopent-3-ene-1,1-dicarboxylate (II,Bn)**

**Table S1:** Screening the ring-closing metathesis conditions employing 0.5mL of **I**

| Run | R | Cat. [mol%, mg] | SnMe_4_ [µL] | *T* [^o^C] | *t* [h] | Conv. [%] |
| --- | --- | --- | --- | --- | --- | --- |
| 1 | Bn | **BM-20%w/w MoOCl_4_@SiO_2-_250** [10, 200] | 200 | 100 | 24 | 17 |
| 2 | Bn | Grubbs 1^st^ generation catalyst [0.5, 5.6] | - | 80 | 24 | >99 |

1. ^1^H NMR spectrum (600 MHz, Methanol-*d*_4_) of **I,Bn** ring-closing metathesis crude reaction catalyzed by BM-20%-MoOCl_4_@SiO_2-250_ (table S1, run 1)

Conversion = $\frac{\boldsymbol{(}\boldsymbol{H}_{\boldsymbol{c}}\boldsymbol{+}\boldsymbol{H}_{\boldsymbol{c'}}\boldsymbol{+}\boldsymbol{H}_{\boldsymbol{d}}\boldsymbol{+}\boldsymbol{H}_{\boldsymbol{d'}}\boldsymbol{)}}{\left( \boldsymbol{H}_{\boldsymbol{c}}\boldsymbol{+}\boldsymbol{H}_{\boldsymbol{c'}}\boldsymbol{+}\boldsymbol{H}_{\boldsymbol{d}}\boldsymbol{+}\boldsymbol{H}_{\boldsymbol{d'}} \right)\boldsymbol{+(}\boldsymbol{H}_{\boldsymbol{c}}\boldsymbol{+}\boldsymbol{H}_{\boldsymbol{c'}}\boldsymbol{+}\boldsymbol{H}_{\boldsymbol{d}}\boldsymbol{+}\boldsymbol{H}_{\boldsymbol{d'}}\boldsymbol{)}}$x100

Conversion = $\frac{\boldsymbol{(}\boldsymbol{0}\boldsymbol{.}\boldsymbol{21}\boldsymbol{)}}{\left( \boldsymbol{0}\boldsymbol{.}\boldsymbol{21} \right)\boldsymbol{+(}\boldsymbol{1}\boldsymbol{.}\boldsymbol{01}\boldsymbol{)}}$x100

Conversion = 17 %

1. ^1^H NMR spectrum (600 MHz, Methanol-*d*_4_) of **I,Bn** ring-closing metathesis crude reaction catalyzed by Grubbs 1^st^ generation catalyst (table S1, run 2)

1. ^1^H NMR spectrum (600 MHz, Methanol-*d*_4_) of isolated **II,Bn**

1. ^13^C NMR (151 MHz, Methanol-*d*_4_) of isolated **II,Bn**

**diethyl 6-oxabicyclo [3.1.0]hexane-3,3-dicarboxylate (A)**

1. ^1^H NMR spectrum (600 MHz, Chloroform-d) of isolated **A**

1. ^13^C NMR (151 MHz, Chloroform-*d*) of isolated **A**

**dibenzyl 6-oxabicyclo[3.1.0]hexane-3,3-dicarboxylate (B)**

1. ^1^H NMR spectrum (300 MHz, Chloroform-d) of isolated **B**

1. ^13^C NMR (151 MHz, CDCl_3_) of isolated **B**

**Section S3: Polymerization**

**S3.1 General polymerization protocol:**

Before use, CPO and functional CPOs (**A** and **B**) required two distinct distillation steps. The **A** and CPO compounds carried out a two-stage drying and distillation protocol. It was first dried with calcium hydride (CaH_2_) overnight, followed by distillation under vacuum. For the second stage, the compound was dried again with sodium hydride (NaH) overnight and subsequently distilled under vacuum. The purification protocol for the **B** compound was modified, it was dried twice with CaH_2_ and distilled at 210 °C under vacuum. After the final distillation was completed, both the **A** and **B** products were collected in the glovebox under an argon atmosphere.

**S3.2 ROCOP with (thio)anhydride/epoxide and ROTERP with (thio)anhydride/epoxide/CS_2_**

Operating in an argon-filled glovebox, the catalyst and the monomers were added to a dried vial equipped with a stir bar. The vial was sealed with a melamine cap containing a Teflon inlay. The sealed vial was then removed from the glovebox and placed in a heating block at the desired temperature for the desired time. At specified points, a sample of the polymerization mixture was cooled to room temperature, and an aliquot was taken for analysis by ^1^H spectroscopy to determine the conversion. At the final reaction point, a sample was taken again to determine the final conversion. After the reaction was completed, the crude polymer mixture was dissolved in 5 mL of DCM and then added dropwise to 30 mL of methanol. This procedure caused the precipitation of the polymer, which was subsequently isolated by centrifugation. This precipitation-isolation cycle was repeated two additional times. The obtained polymer was then dried in a vacuum oven before further analysis.

**S3.3 NMR characterisation**

**S3.3.1 PTA/A ring opening copolymerization**

1. ^1^H NMR spectrum (300 MHz, Chloroform-*d*) of PTA/**A** ROCOP mixture employing LAlRb(OAc)_2_ after 0.5 h (table 2, run 1)

1. ^1^H NMR spectrum (300 MHz, Chloroform-*d*) of PTA/**A** ROCOP mixture employing BEt_3_:PPNCl after 1.5 h (table 2, run 2)

1. ^1^H NMR spectrum (300 MHz, Chloroform-*d*) of PTA/**A** ROCOP mixture employing LAlRb(OAc)_2_ after 0.5 h (table 2, run 7)

1. ^1^H NMR spectrum (300 MHz, Chloroform-*d*) of isolated PTA/**A** ROCOP

1. ^13^C NMR spectrum (126 MHz, Chloroform-*d*) of isolated PTA/**A** ROCOP

1. ^13^C NMR spectrum (126 MHz, Chloroform-*d*) of isolated PTA/**A** ROCOP employing LAlRb(OAc)_2_ (table 2, run 1)

1. ^13^C NMR spectrum (126 MHz, Chloroform-*d*) of isolated PTA/**A** ROCOP employing BEt_3_:PPNCl (table 2, run 2)

1. ^13^C NMR spectrum (126 MHz, Chloroform-*d*) of isolated PTA/**A** ROCOP employing LAlRb(OAc)_2_ (table 2, run 7)

1. 2D NMR HSQC ^1^H-^13^C correlation of of isolated PTA/**A** ROCOP

1. 2D NMR HMBC ^1^H-^13^C correlation of isolated PTA/**A** ROCOP

**S3.3.2 PTA/CPO ring opening copolymerization**

1. ^1^H NMR spectrum (300 MHz, Chloroform-*d*) of PTA/CPO ROCOP mixture employing LAlRb(OAc)_2_ after 3.0 h. (table 2, run 9)

1. ^1^H NMR spectrum (300 MHz, Chloroform-*d*) of isolated PTA/CPO ROCOP

1. ^13^C NMR spectrum (126 MHz, Chloroform-*d*) of isolated PTA/CPO ROCOP

**S3.3.3 PA/A ring opening copolymerization**

1. ^1^H NMR spectrum (300 MHz, Chloroform-*d*) of PA/**A** ROCOP mixture employing LAlRb(OAc)_2_ after 3 h (table 2, run 10)

1. ^1^H NMR spectrum (300 MHz, Chloroform-*d*) of isolated PA/**A** ROCOP

1. ^13^C NMR spectrum (126 MHz, Chloroform-*d*) of isolated PA/**A** ROCOP

1. 2D NMR HSQC ^1^H-^13^C correlation of isolated PA/**A** ROCOP

1. 2D NMR HMBC ^1^H-^13^C correlation of isolated PA/**A** ROCOP

**S3.3.4 CS_2_/A ring opening copolymerization**

1. ^1^H NMR spectrum (300 MHz, Chloroform-*d*) of CS_2_/**A** ROCOP mixture employing LAlRb(OAc)_2_ after 0.5 h. (table 2, run 11)

1. ^1^H NMR spectrum (300 MHz, Chloroform-*d*) of isolated CS_2_/**A** ROCOP

1. ^13^C NMR spectrum (126 MHz, Chloroform-*d*) of isolated CS_2_/**A** ROCOP

1. 2D NMR HSQC ^1^H-^13^C correlation of isolated CS_2_/**A** ROCOP

1. 2D NMR HMBC ^1^H-^13^C correlation of isolated CS_2_/**A** ROCOP

**S3.3.5 PTA/B ring opening copolymerization**

1. ^1^H NMR spectrum (300 MHz, Chloroform-*d*) of PTA/**B** ROCOP mixture employing LAlRb(OAc)_2_ after 0.5 h (table 2, run 12)

1. ^1^H NMR spectrum (300 MHz, Chloroform-*d*) of isolated PTA/**B** ROCOP

1. ^13^C NMR spectrum (126 MHz, Chloroform-*d*) of isolated PTA/**B** ROCOP

1. 2D NMR HSQC ^1^H-^13^C correlation of isolated PTA/**B** ROCOP

1. 2D NMR HMBC ^1^H-^13^C correlation of isolated PTA/**B** ROCOP

**S3.3.6 PTA/B ring opening copolymerization**

1. ^1^H NMR spectrum (300 MHz, Chloroform-*d*) of PA/**B** ROCOP mixture employing LAlRb(OAc)_2_ after 7.0 h (table 2, run 13)

1. ^1^H NMR spectrum (300 MHz, Chloroform-*d*) of isolated PA/**B** ROCOP

1. ^13^C NMR spectrum (126 MHz, Chloroform-*d*) of isolated PA/**B** ROCOP

1. 2D NMR HSQC ^1^H-^13^C correlation of isolated PA/**B** ROCOP

1. 2D NMR HMBC ^1^H-^13^C correlation of isolated PA/**B** ROCOP

**S3.3.7 Ring opening terpolymerization (ROTER)**

**Table S1** PTA, PA and CS_2_ with **A** ROTERP employing LAlRb(OAc)_2_

| run | Experiment | Ratio | *t*  [h] | Conv. [%]*^c^* | *M*_n_ [kg/mol] (*Đ*)*^d^* | T_g, onset_*^e^*  [°C] | T_d, 5%_*^f^*  [°C] |
| --- | --- | --- | --- | --- | --- | --- | --- |
| 1*^a^* | **A**/PTA/PA | 1000:250:250 | 3.00 | >99 (PA) | 30.5 (1.2) | 62 | 292 |
| 2*^b^* | **A**/PTA/CS_2_ | 500:250:500 | 1.25 | >99 (PTA) | 35.6 (2.0) | 58 | 228 |

ROCOPs conducted with 1 eq. catalysts at *^a^*100 °C and *^b^*80 °C. *^c^* Relative integral of aromatic resonances from residual PTA or PA versus polymer in the normalised ^1^H NMR spectrum of final aliquot. *^d^* Determined by GPC (gel permeation chromatography) measurements conducted in THF, using narrow polystyrene standards to calibrate the instrument. *^e^* Determined by DSC. *^f^* Determined by TGA.

**A/PTA/PA ring opening terpolymerization**

1. ^1^H NMR spectrum (300 MHz, Chloroform-*d*) of **A**/PTA/PA ROTERP mixture employing LAlRb(OAc)_2_ from 0 to 3 h (table S1, run 3) comparing with PTA/**A** and PA/**A** ROCOP

1. ^1^H NMR spectrum (300 MHz, Chloroform-*d*) of isolated **A**/PTA/PA ROTERP

1. ^13^C NMR spectrum (126 MHz, Chloroform-*d*) of isolated **A**/PTA/PA ROTERP

1. 2D NMR HSQC ^1^H-^13^C correlation of isolated **A**/PTA/PA ROTERP

1. 2D NMR HMBC ^1^H-^13^C correlation of isolated **A**/PTA/PA ROTERP

**A/PTA/CS_2_ ring opening terpolymerization**

1. ^1^H NMR spectrum (300 MHz, Chloroform-*d*) of **A**/PTA/CS_2_ ROTERP mixture employing LAlRb(OAc)_2_ from 0 to 1.25 h (table S1, run 1) comparing with PTA/**A** and CS_2_/**A** ROCOP

1. ^1^H NMR spectrum (300 MHz, Chloroform-*d*) of isolated **A**/PTA/CS_2_ ROTERP

1. ^13^C NMR spectrum (126 MHz, Chloroform-*d*) of isolated **A**/PTA/CS_2_ ROTERP

1. 2D NMR HSQC ^1^H-^13^C correlation of isolated **A**/PTA/CS_2_ ROTERP

1. 2D NMR HMBC ^1^H-^13^C correlation of isolated **A**/PTA/CS_2_ ROTERP

**Section S4: GPC, DSC and TGA Characterisation**

**S4.1 GPC characterization**

**
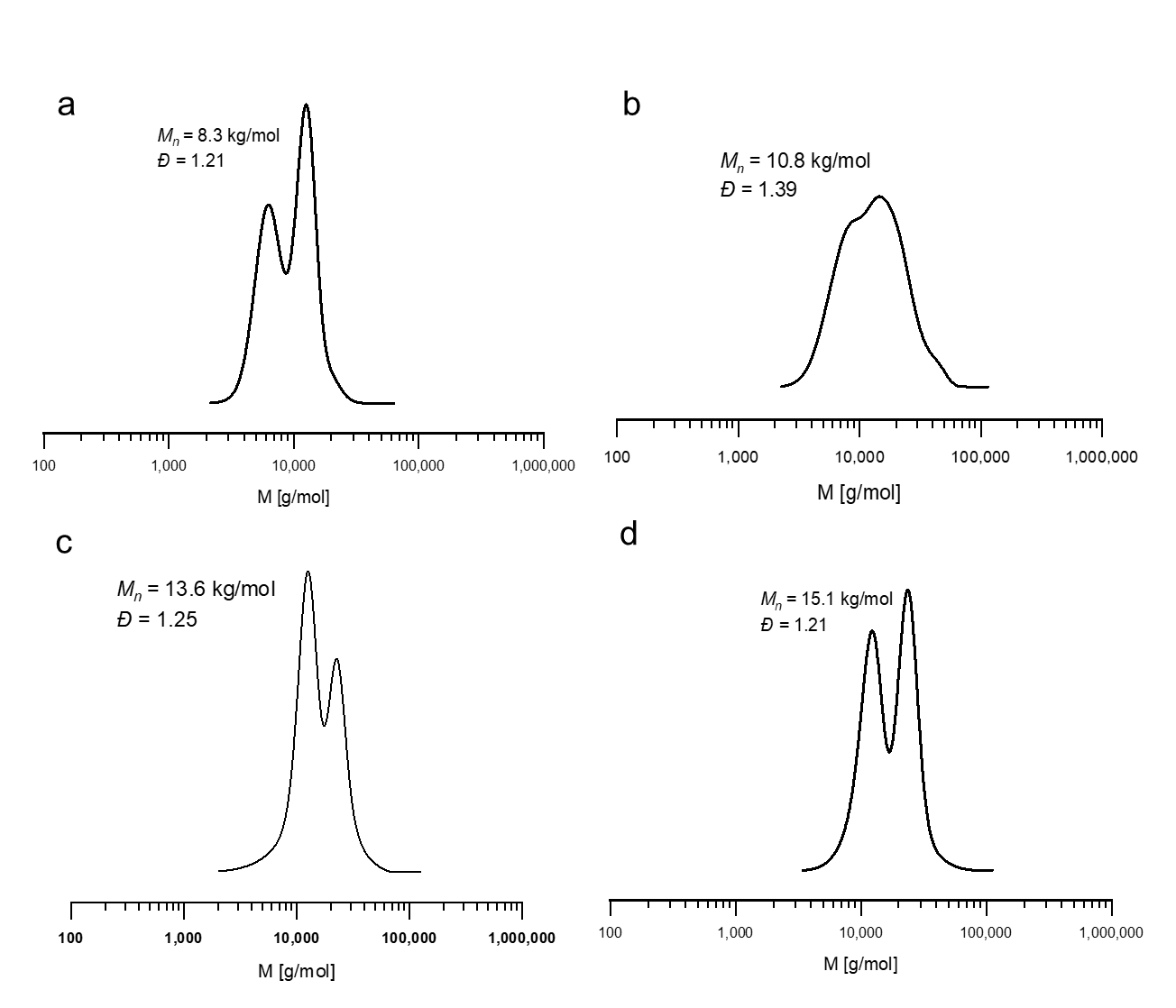
**

**
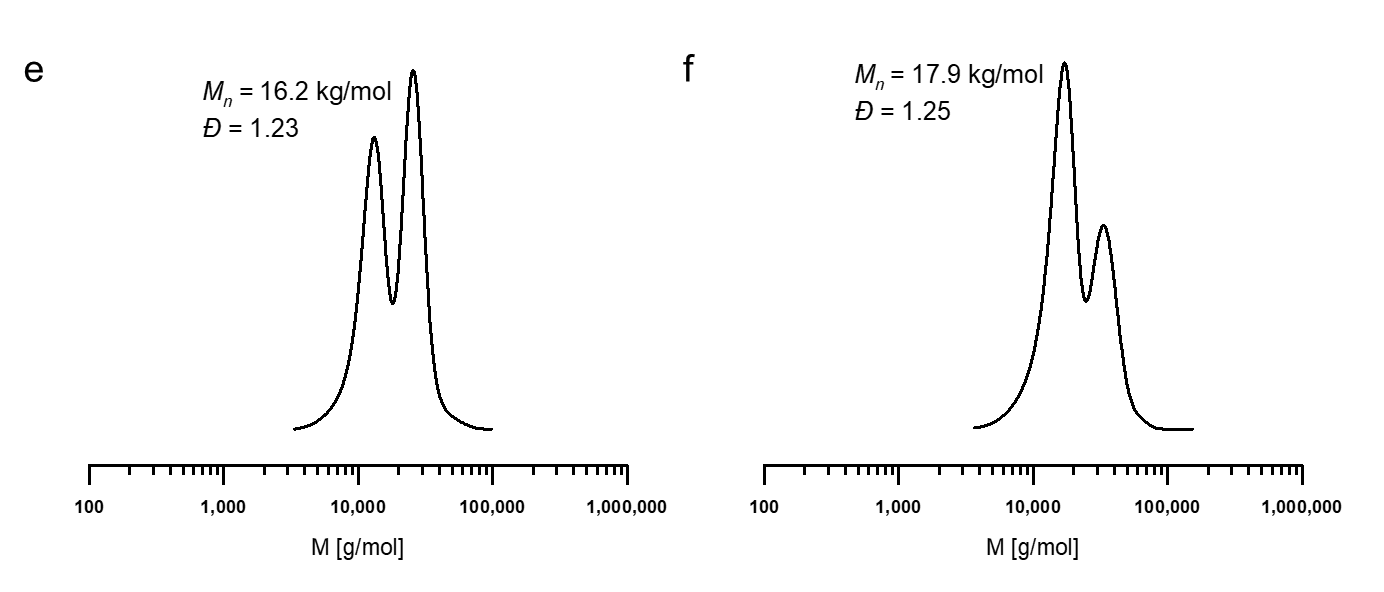
**

**
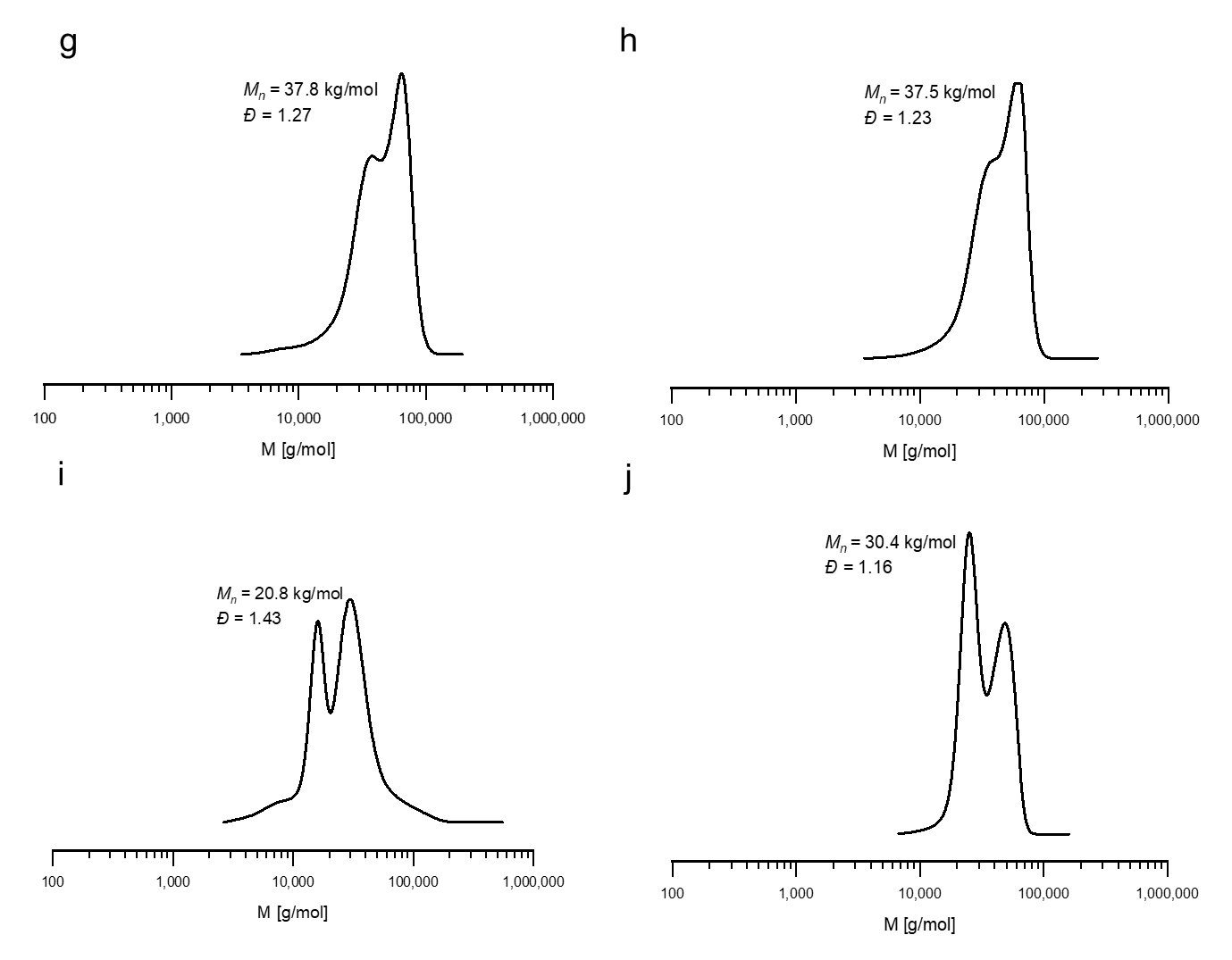
**

**
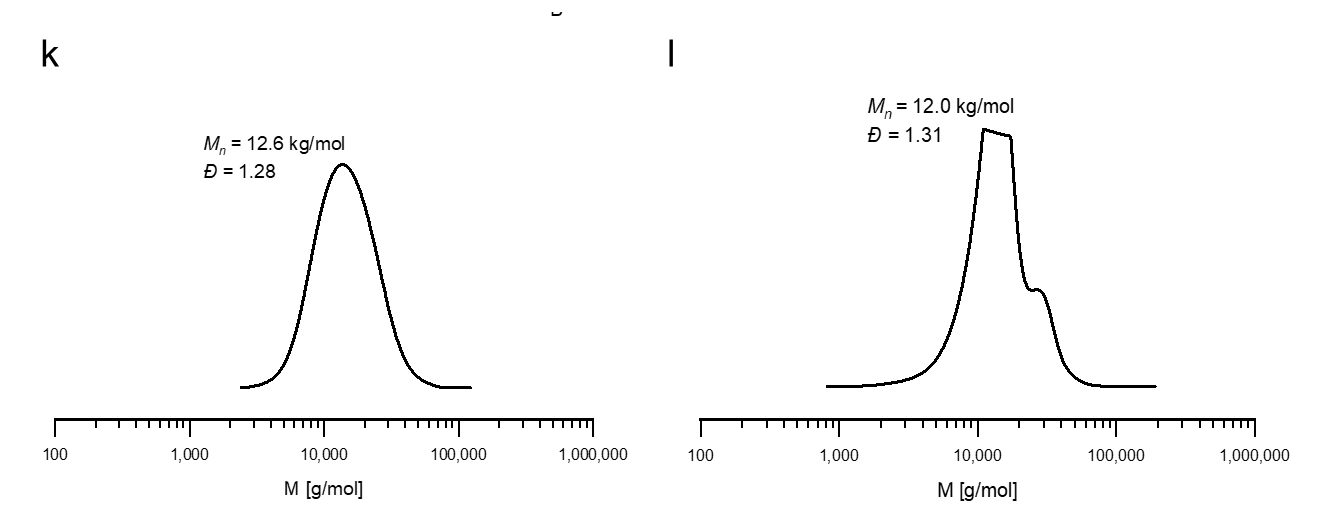
**

**
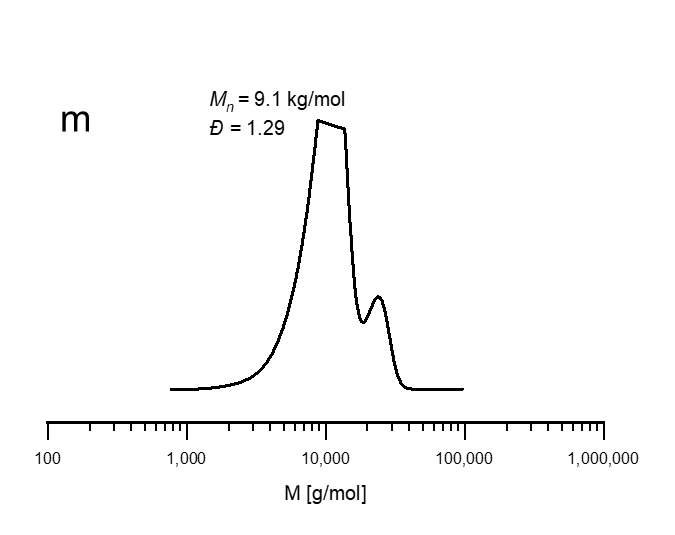
**

1. GPC traces of isolated ROCOP in table 2 a) run 1, b) run 2, c) run 3, d) run 4, e) run 5, f) run 6, g) run 7, h) run 8, i) run 9, j) run 10, k) run 11, l) run 12, and m) run 13

**S4.2 DSC and TGA characterisation**

**
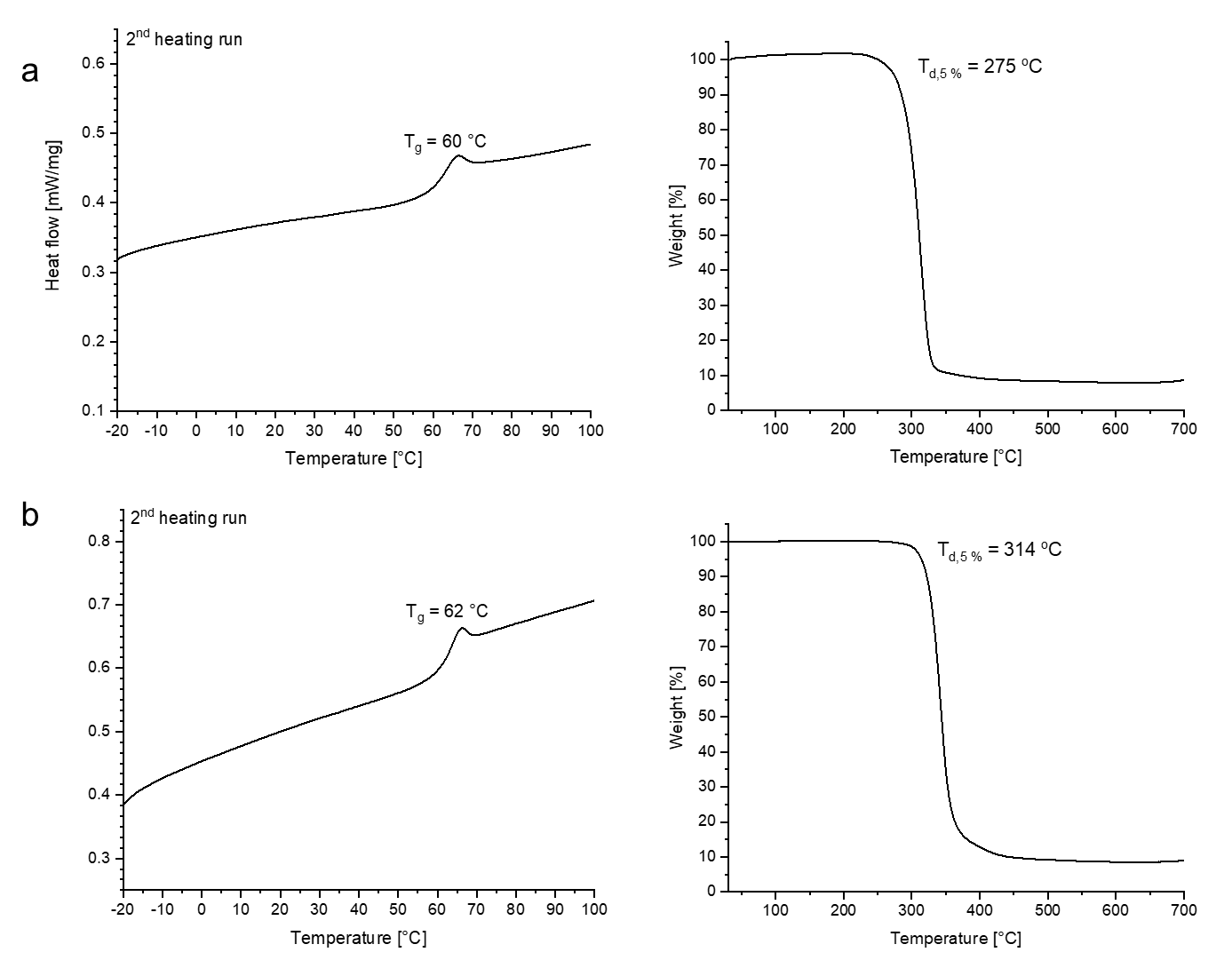

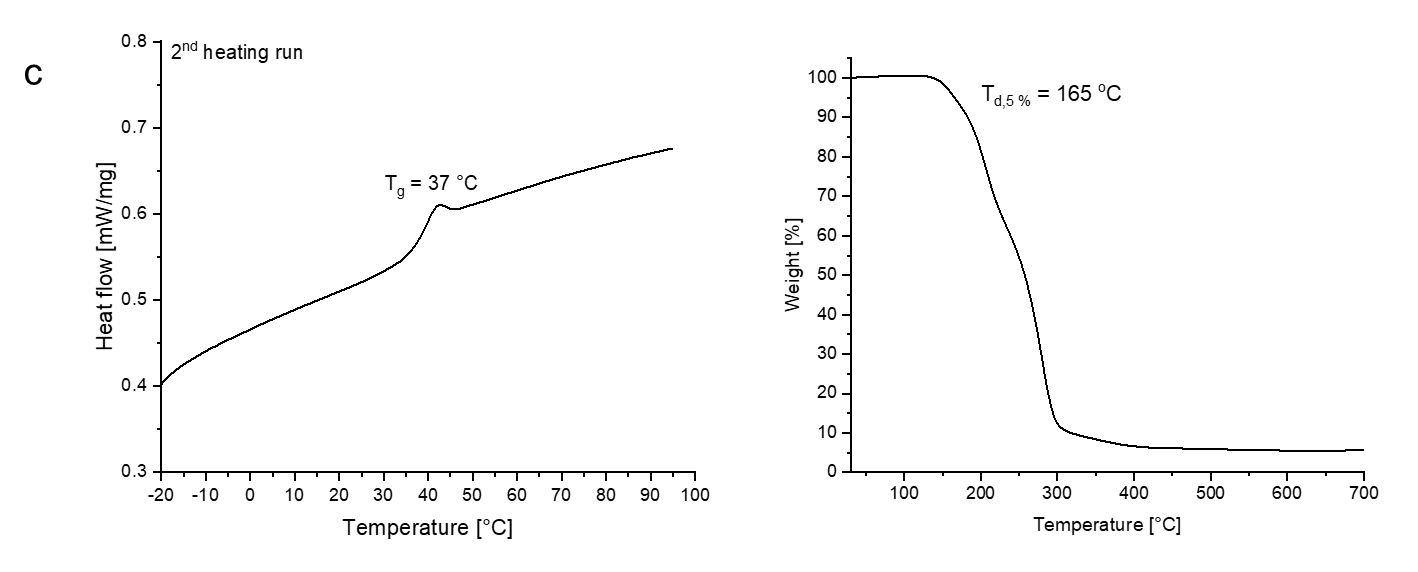
**

**
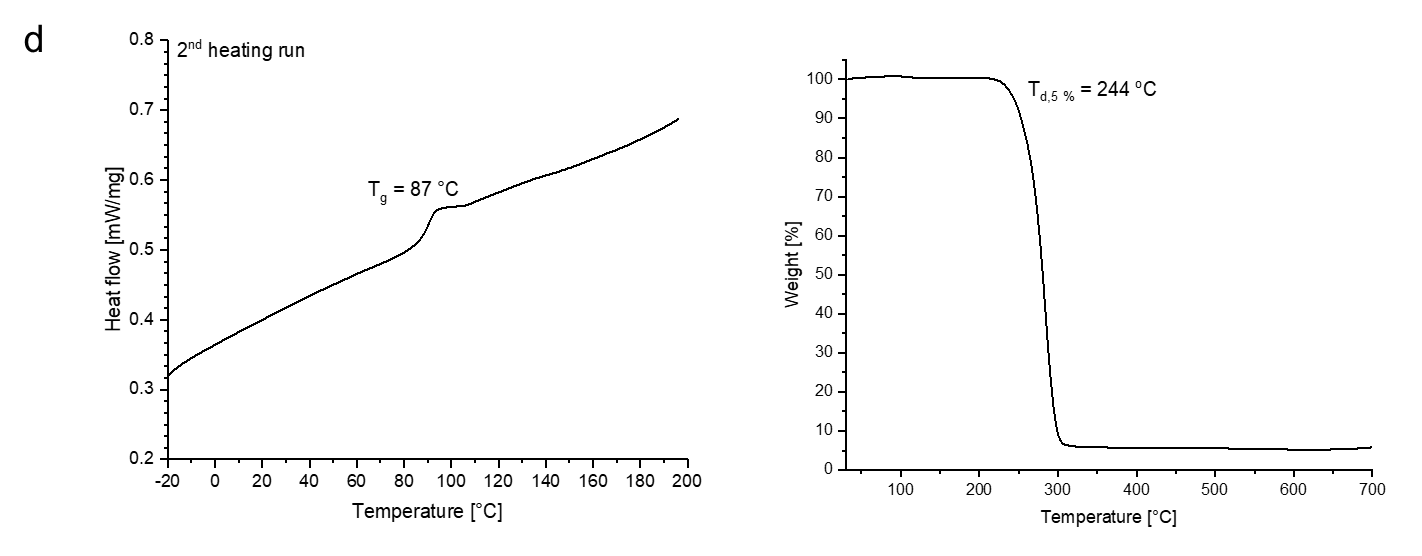
**

**
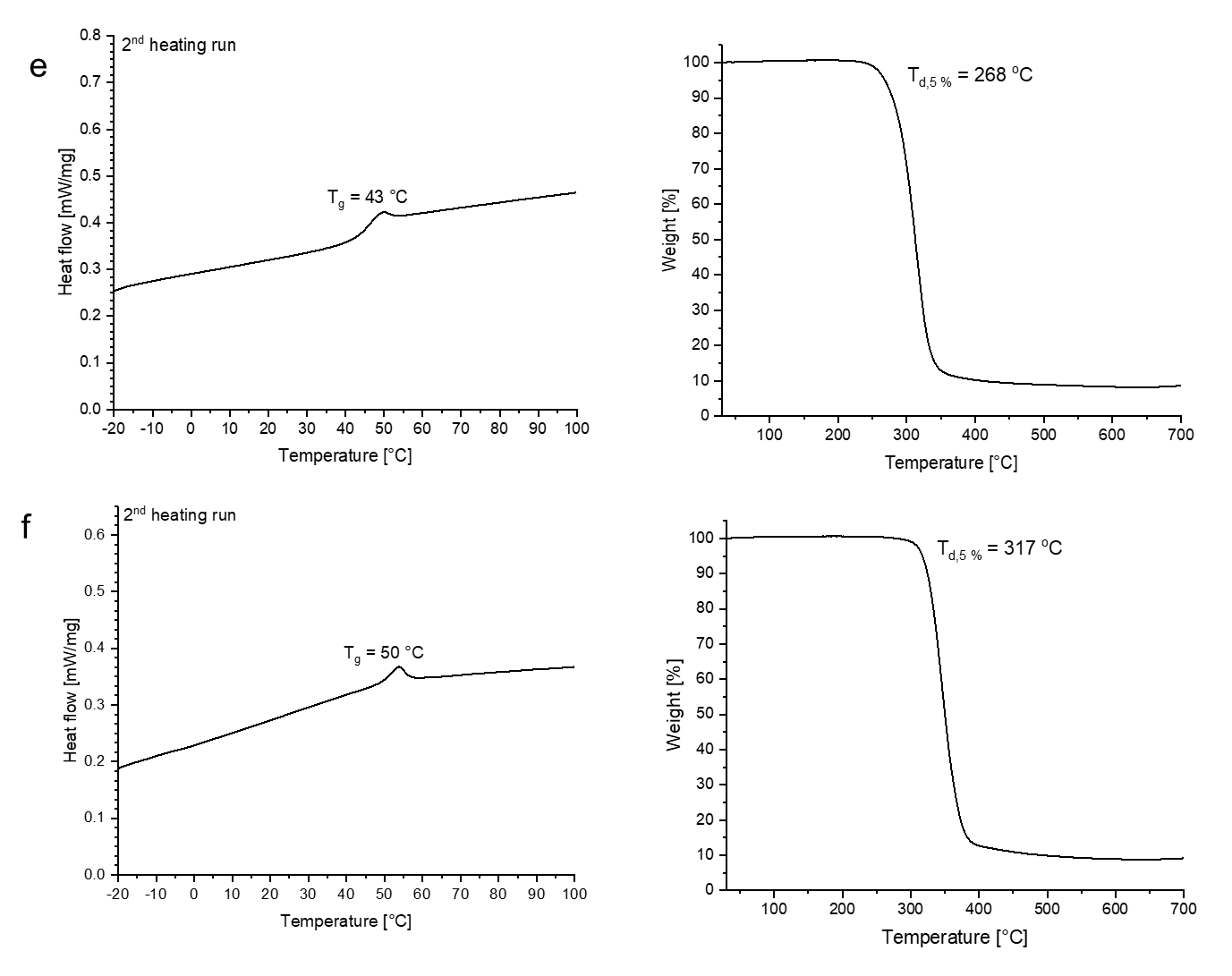
**

1. DSC heating curve and TGA curve of a) isolated **A**/PTA ROCOP (table 2, run 7) b) isolated **A**/PA ROCOP (table 2, run 10), c) isolated **A**/CS_2_ ROCOP (table 2, run 11), d) isolated CPO/PTA ROCOP (table 2, run 9), e) isolated **B**/PTA ROCOP (table 2, run 12) and f) isolated **B**/PA ROCOP (table 2, run 12)

**
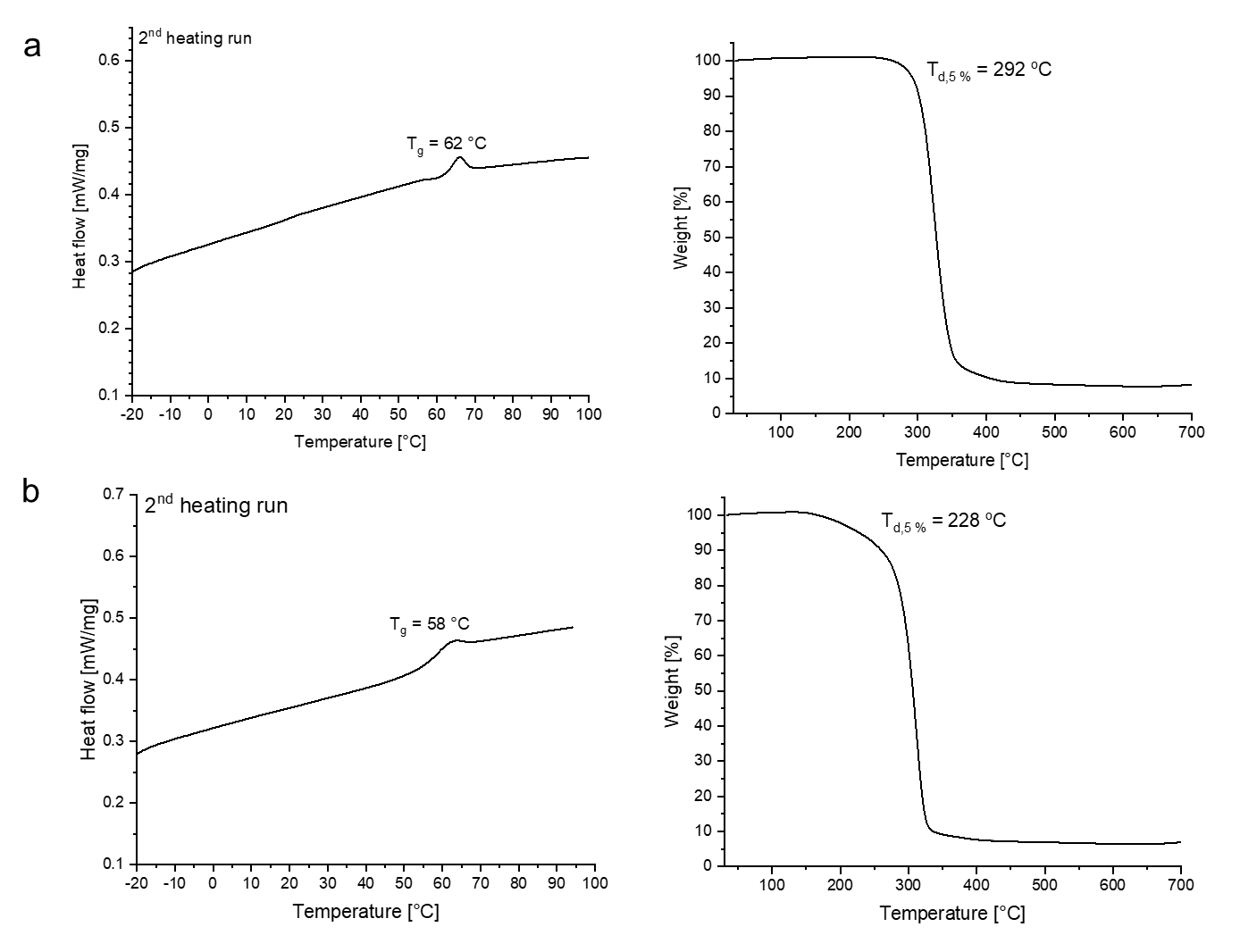
**

1. DSC heating curve and TGA curve of a) isolated **A**/PTA/PA ROTERP (table S1, run 1) and b) isolated **A**/PTA/CS_2_ ROTERP (table S1, run 2)

**Section S5: Degradation studies**

The degradation was studied by following literature^5, 6^, 60 mg of polymer sample was weighed into a vial containing a magnetic stirrer. 5 mL of the respective degradation solution was added into the vial. The vials were then placed in a heating block at 40 °C for 7 days. 5 mL of 1 M HCl was used for acid hydrolysis. 5 mL of 5 wt.% NaOH in 60% ethanol solution was used for alkaline hydrolysis. 5 mL of 7 M ammonia in methanol was used for aminolysis. 5 mL of 30%w/v hydrogen peroxide was used for oxidative degradation. After degradation, the polymer sample was filtered, washed twice and dried to determine the weight loss percentage.

1. ^1^H NMR spectrum (300 MHz, Chloroform-*d*) of isolated **A**/PTA ROCOP before and after aminolysis

**Section S6: Matrix-Assisted Laser Desorption/Ionization Time-of-Flight) mass (MALDI-TOF)**


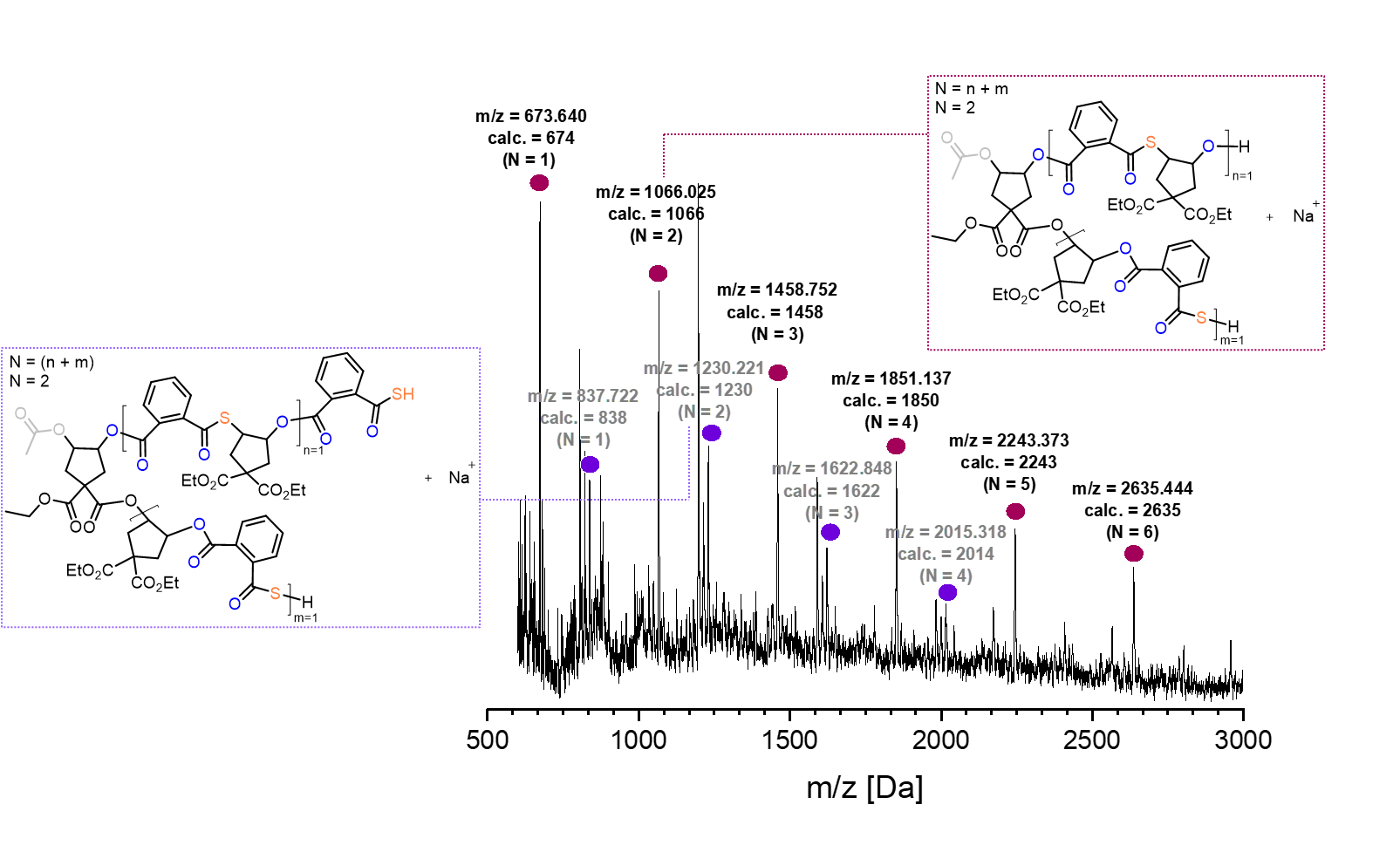


1. MALDI-TOF of isolated **A**/PTA ROCOP with 1:25:25 ratio of LAlRb(OAc)_2_ cat: **A**: PTA

**Section S7: Copolymer with chain transfer agent (CTA)**

20 eq. of 1,4-benzenedimethanol was used as chain transfer agent (CTA) of ring opening copolymerization of **A**/PTA with 1:1000:500 ratio (LAlRb(OAc)_2_ cat: **A**: PTA).

1. GPC traces of isolated **A**/PTA ROCOP with 20 eq. of chain transfer agent (CTA), blue line) and without CTA, black line

References

(1) Manjunatha, B. R.; Stühler, M. R.; Quick, L.; Plajer, A. J. Improved access to polythioesters by heterobimetallic aluminium catalysis. *Chemical Communications* **2024**, *60* (34), 4541-4544, 10.1039/D4CC00811A. DOI: 10.1039/D4CC00811A.

(2) Stühler, M. R.; Gallizioli, C.; Rupf, S. M.; Plajer, A. J. Ring-opening terpolymerisation of phthalic thioanhydride with carbon dioxide and epoxides. *Polymer Chemistry* **2023**, *14* (42), 4848-4855, 10.1039/D3PY01022H. DOI: 10.1039/D3PY01022H.

(3) Tangyen, N.; Natongchai, W.; Del Gobbo, S.; D’Elia, V. Revisiting the Potential of Group VI Inorganic Precatalysts for the Ethenolysis of Fatty Acids through a Mechanochemical Approach. *ACS Omega* **2024**, *9* (17), 19712-19722. DOI: 10.1021/acsomega.4c02190.

(4) Nečas, D.; Turský, M.; Tišlerová, I.; Kotora, M. Nickel-catalyzed cyclization of α,ω-dienes: formation vs. cleavage of C–C bonds. *New Journal of Chemistry* **2006**, *30* (5), 671-674, 10.1039/B601631F. DOI: 10.1039/B601631F.

(5) Liu, Y.; Bejjanki, N. K.; Jiang, W.; Zhao, Y.; Wang, L.; Sun, X.; Tang, X.; Liu, H.; Wang, Y. Controlled Syntheses of Well-Defined Poly(thionophosphoester)s That Undergo Peroxide-Triggered Degradation. *Macromolecules* **2019**, *52* (11), 4306-4316. DOI: 10.1021/acs.macromol.9b00061.

(6) Manjunatha, B. R.; Sengoden, M.; Stühler, M. R.; Langer, R.; Darensbourg, D. J.; Plajer, A. J. Monomer-Dependent Selectivity in Sulfur-Containing Ring-Opening Copolymerisation: Bimetallic Catalysis for Predictive Design of Degradable Polymers. *Angewandte Chemie International Edition* **2025**, *64* (42), e202508985. DOI: 10.1002/anie.202508985.
